# Efficacy and safety of percutaneous microwave ablation for liver tumors using an antenna with anti-phase technology offering ultraspherical ablation

**DOI:** 10.2478/raon-2025-0064

**Published:** 2025-12-16

**Authors:** Erbil Arik, Onur Taydas, Tunahan Dertli, Omer Faruk Sevinc, Ahmet Burak Kara, Omer Faruk Topaloglu, Mustafa Ozdemir, Adem Senturk, Alp Omer Canturk, Ilhan Hacibekiroglu, Mehmet Halil Ozturk

**Affiliations:** 1 Marmara University, Faculty of Medicine, Department of Radiology, istanbul, Turkey; 2 Sakarya University, Faculty of Medicine, Department of Radiology, Sakarya, Turkey; 3 Kocaeli City Hospital, Department of Radiology, Kocaeli, Turkey; 4 Gaziantep City Hospital, Department of Radiology, Gaziantep, Turkey; 5 Sakarya University, Faculty of Medicine, Department of General Surgery, Sakarya, Turkey; 6 Sakarya University, Faculty of Medicine, Department of Medical Oncology, Sakarya, Turkey

**Keywords:** hepatocellular carcinoma, liver metastasis, thermal ablation, microwave ablation, ultraspherical ablation

## Abstract

**Background:**

Anti-phase technology, a novel advancement in microwave antennas for percutaneous liver ablations, forms more spherical ablation zones. This study aimed to evaluate the efficacy and safety of microwave ablation (MWA) treatment for liver tumors using a microwave antenna equipped with anti-phase technology.

**Patients and methods:**

The study included 92 patients (133 lesions) treated with MWA for hepatocellular carcinoma (HCC) or liver metastases. Of these, nine patients had HCC, and 83 had metastases (46 colorectal and 37 non-colorectal metastases). Retrospective analysis was conducted on patients’ age, sex, pre- and post-procedural laboratory values (white blood cell count, neutrophil-to-lymphocyte ratio), tumor and ablation zone dimensions (preprocedure and post-procedure day 1 and months 1, 3, and 6), details of the single-shot MWA procedure (duration, power output), procedure-related complications, and local progression/recurrence during follow-up.

**Results:**

The technical success rate of MWA was 100%. Ablations were performed at a median power output of 80 watts (range: 50–100), and the mean ablation duration was 5.2 ± 2.1 minutes. Follow-up imaging revealed an ablation zone diameter-to-tumor diameter ratio of 1.63 ± 0.3. Major complications occurred in three patients (3.2%) and included liver abscess (n = 1/92), hemorrhage (n = 1/92), and pleural effusion (n = 1/92). Minor complications were observed in 29 patients (31.5%). The median follow-up time of the patients was 33 (range 10–36) months. The median disease-free survival time was 25 months (95% confidence interval: 21–27). During the 24-month follow-up, local tumor progression occurred in 39 patients (42.4%). Tumor size was identified as an independent risk factor for local progression (p = 0.012).

**Conclusions:**

This study represents the longest follow-up duration and the largest patient cohort for the MWA treatment of liver tumors using anti-phase technology. The results demonstrated high technical success and acceptable local control and complication rates.

## Introduction

Hepatocellular carcinoma (HCC) accounts for 85% of primary liver malignancies and is the third leading cause of cancer-related organ-specific mortality globally. If left untreated, the five-year survival rate is approximately 5–9%. The liver is also a common site for distant metastases, particularly from the gastrointestinal system.^1,2^ Surgical resection is the standard treatment for early- and very early-stage HCC or liver metastases. However, only 10–20% of patients qualify for resection or transplantation due to factors such as advanced cirrhosis, insufficient liver function, multifocal or advanced disease, tumor location, portal venous invasion, and comorbidities. Surgical treatment is associated with a 2–5% mortality rate and a 20% morbidity rate.^1,3^ For patients with HCC or metastatic liver tumors who are unsuitable for surgery, interventional treatments such as transarterial therapies and thermal ablation (TA) methods are available. Transarterial therapies involve selective delivery of chemotherapeutic agents (transarterial chemoperfusion, chemoembolization, and embolization) or radioactive materials (radioembolization) to the liver. TA methods include radiofrequency ablation (RFA), microwave ablation (MWA), laser-induced interstitial thermotherapy, and cryoablation.^2^ These methods can be used as standalone treatments or as bridging therapies before liver transplantation.^1^

RFA and MWA are the most commonly used TA techniques. Both aim to deliver heat to malignant tissues, raising the temperature above 60°C to induce coagulation necrosis. Like surgical margins, complete ablation targets the tumor and a surrounding 5–10 mm margin of healthy tissue.^4,5^

RFA, which has been in use longer, generates frictional heating by ionic agitation using alternating current. However, RFA has two significant limitations. The first is the boiling and charring that occur in tissue at temperatures of 100°C and above. The gas or charred tissue formed can obstruct heat conduction, creating unwanted insulation. The second limitation is the “heat-sink” effect, in which vascular structures near the ablation zone dissipate the heat from this area. These factors may prevent the tissue from reaching the necessary temperature, leading to incomplete ablation.^3^ MWA, on the other hand, is a newer method that relies on heating water molecules through an electromagnetic field oscillating at frequencies of 900 or 2,450 MHz. This frictional heating increases the temperature in all the tissue around the antenna to a different degree. Compared to RFA, MWA offers several theoretical advantages due to using electromagnetic fields instead of electrical energy. Due to its physical principle, microwave ablation (MWA) is less affected by the heat-sink effect and tissue charring and, therefore, less influenced by heat insulation caused by these factors.^6^ As a result, it can achieve higher intratumoral temperatures, leading to larger and more homogeneous ablation zones in a shorter time.^3,7^ Furthermore, multiple antennas can be used to create larger ablation zones.^8^ However, MWA presents specific challenges, including difficulties achieving safe and effective power distribution, larger antenna diameters, and an increased risk of overheating at the antenna tip and connecting cable.^9^ Since its coming into use, various innovations in technique and device technology have been developed to enhance the efficacy of MWA.

A microwave antenna consists of a coaxial cable, which includes an inner conductor, dielectric material, and an outer conductor. In microwave antennas, leaking currents can result in backward heating, which causes the ablation zone, ideally shaped as ellipsoidal or spherical, to become comet-shaped. The tail of this comet shape represents the ablation of normal tissue located behind the active tip of the antenna. Technological developments have addressed this issue by incorporating slots, chokes, or sleeves in the antenna, installing cooling systems, optimizing the outer conductor structure, or adjusting the electromagnetic energy frequency.^10^ The recently introduced anti-phase technology integrates these advancements by incorporating a mechanism within the antenna to create opposing-phase microwave radiation. It suppresses backward currents at the edge of the ablation zone, preventing backward heating and enabling more spherical (ultraspherical) ablation. This microwave antenna technology has been addressed in limited ex-vivo, in-vivo preclinical, and in-vivo clinical current studies.^11-14^ To the best of our knowledge, our study has the largest number of patients and lesions among in-vivo clinical studies. This study aimed to investigate liver tumor ablation’s success, efficacy, and safety using microwave antennas equipped with anti-phase technology.

## Patients and methods

**FIGURE 1.**
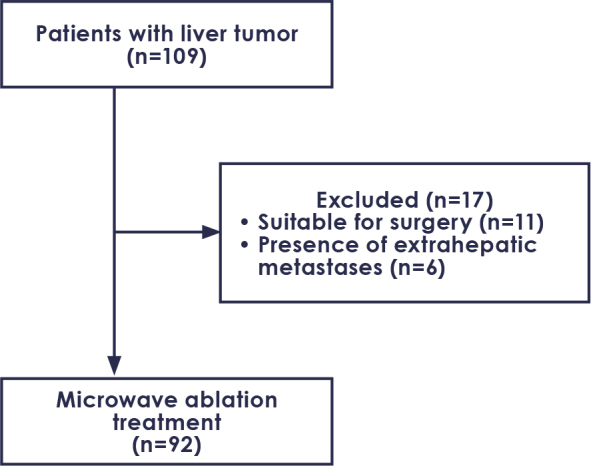
Flowchart of the patients.

This retrospective, cross-sectional, single-center study was conducted in compliance with the principles of the Declaration of Helsinki after receiving ethical approval (number: E-71522473-050.04-349246-63/26.03.2024). Patients who underwent thermal liver ablation at our clinic between January 2019 and March 2024 were included (Figure 1). A multidisciplinary tumor board made all decisions regarding MWA treatment. The inclusion and exclusion criteria are summarized in Table 1.

**TABLE 1.** Inclusion and exclusion criteria of the study

Included	Excluded
Lesions not amenable to surgical treatment	Presence of extrahepatic metastases
Insufficient functional liver capacity after surgery	Suspected pregnancy
Patients unable to undergo general anesthesia due to comorbidities	Uncorrectable coagulopathy
Cases with the use of the particular antenna technology	Any other transarterial or percutaneous treatment

Retrospective analysis was conducted on patients’ age, sex, pre- and post-procedure laboratory values (white blood cell count, neutrophil-to-lymphocyte ratio), tumor dimensions (pre-procedure and post-procedure day 1 and months 1, 3, and 6), details of the MWA procedure (duration, power output), complications, and local progression or recurrence during follow-up.

### Procedures

An interventional radiologist with 12 years of experience in interventional oncology performed all procedures. Prophylactic antibiotics were administered to all patients before the procedure. MWA was performed under deep sedation and analgesia under the supervision of an anesthesiologist. Local anesthesia with 10 cc of prilocaine (Priloc 2%, Vem Pharmaceuticals, Turkey) was applied to the subcutaneous region and liver capsule under sterile conditions.

**FIGURE 2.**
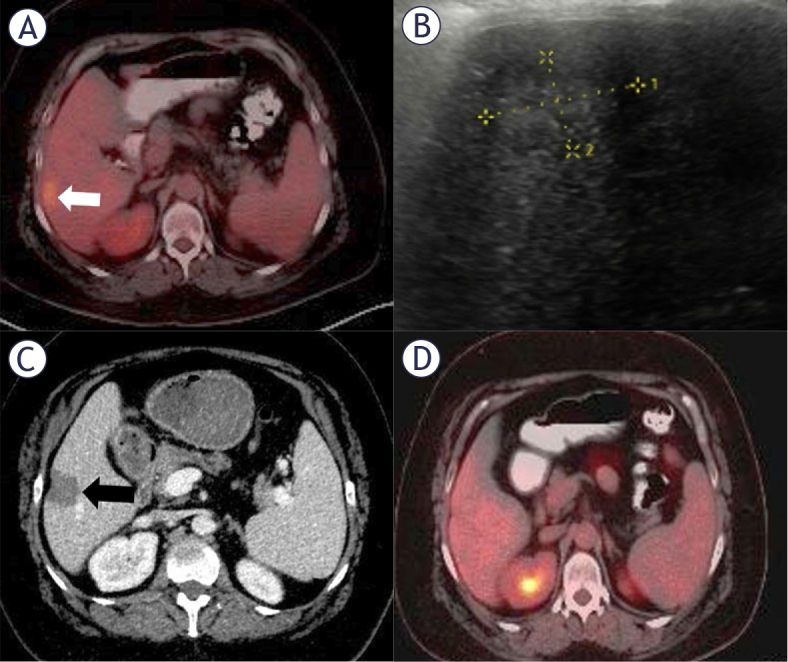
**(A-D)** Radiological findings in a 48-year-old woman with ductal breast carcinoma. PET-CT revealed a hypermetabolic metastasis in segment 6, **(A)** (white arrow). Ultrasound showed a subcapsular hypoechoic lesion **(B),** which was treated with percutaneous microwave ablation under ultrasound guidance. Follow-up CT one day after ablation demonstrated a lesion/ablation area ratio of 1.6 without contrast enhancement, **(C)** (black arrow). PET-CT at 36 months showed no pathological FDG uptake in the liver **(D)**.

Lesions were accessed under ultrasound guidance using a convex probe (V8, Samsung, Republic of Korea). For MWA, a 15-gauge Dophi™ M150E antenna (Surgnova, China) was utilized, operating at a frequency of 2.45 GHz with a power output of 50–100 watts. This shaft-cooled antenna with continuous energy transfer was equipped with anti-phase technology (Figure 2A–D). Ablation parameters, including power and duration, were adjusted based on the manufacturer’s ablation chart according to the lesion size. For 16 patients (17.4%), additional hydrodissection with 5% dextrose solution was performed for lesions within 5 mm of bowel loops, the diaphragm, or major vessels.

For all patients, the ratio of the post-procedure ablation zone diameter to the pre-procedure tumor diameter was calculated based on contrast-enhanced CT images obtained the day after the procedure (64-slice multidetector CT, Aquilion 64; Toshiba Medical Systems, USA). All measurements were made manually based on the diameter of the longest axes before the procedure and after a single ablation session. Follow-up imaging was performed every three months during the first year and biannually thereafter, using CT, MRI, or positron emission tomography-CT (PET-CT) scans. Imaging findings were assessed using the modified Response Evaluation Criteria in Solid Tumors to determine the response. During follow-up, local tumor progression (LTP) was defined as detecting nodular enhancement adjacent to the ablation zone.

### Definitions

The target ablation zone was defined as the ablation of the tumor with a 10-mm safety margin while preserving normal parenchyma and nontarget tissues. Technical success was defined as complete ablation of the target lesion as confirmed by contrast-enhanced CT one day after the procedure. Complications were classified as minor or major according to the Society of Interventional Radiology reporting standards.^15^ Minor complications required only observation without treatment or hospitalization, while major complications necessitated prolonged hospitalization, an unplanned increase in patient care level, or resulted in squeal or death.

### Statistical analysis

MedCalc (version 12, Ostend, Belgium) was used for statistical analyses. Descriptive statistics were presented as median (minimum-maximum) and mean ± standard deviation values. Categorical variables were expressed as frequencies and percentages. Fisher, Pearson’s chi-squared, and Yates’ corrected version of Pearson’s chi-squared tests were used to compare categorical variables. The independent-sample t-test was used for the comparison of continuous variables with a normal distribution, and the Mann-Whitney U test was for the data that did not conform to the normal distribution according to the Kolmogorov-Smirnov test. Univariate and multivariate Cox proportional hazards models were utilized to identify risk factors for LTP. The Kaplan-Meier analysis was used to evaluate disease-free survival analysis. A p-value of < 0.05 was accepted as statistically significant.

## Results

A total of 92 patients who underwent MWA treatment for HCC or liver metastases were included in the study. The procedure was performed on a total of 133 lesions. The mean age of the patients was 59.8 ± 12.3 years; 49 were female (53.3%), while 43 were male (46.7%). The mean white blood cell count was 6.5 ± 2.4 ×10^3^/μL, and the median neutrophil-to-lymphocyte ratio was 2.6 (range: 1–10.4). The mean tumor size was 26.1 ± 13.4 mm. The clinical and demographic characteristics of the patients are summarized in Table 2.

**TABLE 2.** Clinical and demographic characteristics of the patients

	n
Sex (female/male)	49 (53.3%)/43 (46.7%)
Age	59.8 ± 12.3
Age group (≤ 65/> 65 years)	60 (65.2%)/32 (34.8%)
White blood cell count	6.5 ± 2.4 ×10^3^/L
White blood cell count group (≤ 8 ×10^3^ /L / > 8 ×10^3^/L)	70 (76.1%)/22 (23.9%)
Neutrophil-to-lymphocyte ratio	2.6 (range: 1-10.4)
Neutrophil-to-lymphocyte ratio group (≤ 2 / > 2)	23 (25%)/69 (75%)
Tumor location (favorable/unfavorable)	109 (81.9%)/24 (18.1%)
Tumor size	26.1 ± 13.4 mm
Tumor size group (≤ 3 cm / >3 cm)	93 (69.9%)/40 (30.1%)

Of the 92 patients, nine (9.8%) were diagnosed with HCC, and 83 (90.2%) had metastases. Among patients with metastases, 46 (55.4%) had colorectal metastases, and 37 (44.6%) had non-colorectal metastases (Table 3).

**TABLE 3.** Primary tumors of patients who underwent ablation due to liver metastasis

	n	%
**Colorectal**		
Colon	32	38.5
Rectum	14	16.9
**Non-colorectal**		
Breast	14	16.9
Gastric	8	9.6
Pancreas	5	6
Ova rian	5	6
Lung	3	3.6
Endometrium	2	2.5

MWA achieved the targeted ablation zone in all patients, resulting in a technical success rate of 100%. A median power of 80 watts (50–100 range) was applied to the lesions, and the mean ablation duration was 5.2 ± 2.1 minutes. The mean ratio of the ablation zone area to the tumor area was 1.63 ± 0.3.

Minor complications occurred in 29 patients (31.5%), including post-procedural pain (n = 15), subfebrile fever (n = 8), and fatigue (n = 6). All minor complications were resolved within 48 hours. Major complications were observed in 3 patients (3.2%). They included a liver abscess requiring drainage (n = 1), hemorrhage necessitating embolization eight hours after the procedure (n = 1), and pleural effusion requiring drainage two hours after the procedure (n = 1).

**FIGURE 3.**
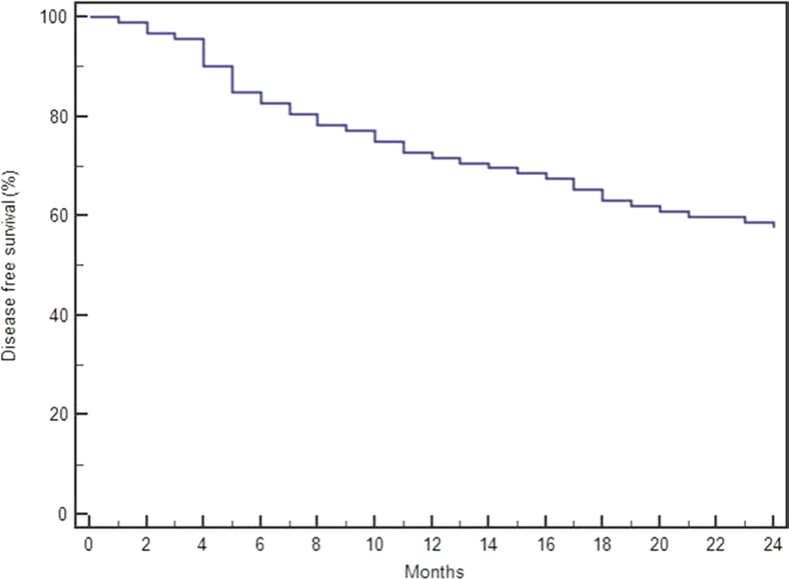
Analysis of disease-free survival (DFS) in patients who underwent microwave ablation (MWA).

The median follow-up time of the patients was 33 (range 10–36) months. According to the Kaplan-Meier analysis, the third-, sixth-, 12th-, and 24th-month disease-free survival (DFS) rates were 95.6%, 82.6%, 72.8%, and 57.6%, respectively. The median DFS time was 25 months (95% confidence interval: 21–27) (Figure 3).

During the 24-month follow-up, local tumor progression was observed in 39 patients (42.4%). Univariate analysis identified tumor size as the only independent risk factor for LTP (p = 0.012) (Table 4). In a multivariate analysis, tumor size was categorized as ≤3 cm, 3–5 cm, and ≥5 cm. It was determined to be an independent risk factor for local tumor progression (LTP), with a hazard ratio of 1.733 (95% CI: 1.541–2.873) and a p-value of 0.037.

**TABLE 4.** Univariate analysis of factors associated with local tumor progression in MWA-treated patients

Variables	p
Sex (male *vs*. female)	0.372
Age (≤ 65 *vs. > 65* years)	0.415
White blood cell count (≤8 ×10^3^/L *vs. > 8* ×10^3^/L)	0.554
Neutrophil-to-lymphocyte ratio (≤2 *vs*. > 2)	0.297
Primary tumor (HCC *vs*. metastasis)	0.624
Metastasis type (colorectal *vs*. non-colorectal)	0.198
Tumor location (favorable *vs*. unfavorable)	0.339
Tumor size (≤3 cm *vs*. > 3 cm)	0.012

1 HCC = hepatocellular carcinoma; MWA = microwave ablation

## Discussion

The most notable feature of our study is its focus on the use of an MWA antenna with anti-phase technology offering ultraspherical ablation, making it, to our knowledge, the second study in vivo on humans and the largest to date, with the longest follow-up period. In addition, unlike the previous clinical study involving the same antenna, the lesions treated in our research predominantly included metastatic lesions, with a significant proportion originating from colorectal cancer.

The principle underlying MWA involves the application of an electromagnetic field at frequencies ranging from 900 to 2,500 MHz to achieve lethal temperatures within tissues, resulting in tissue destruction. Microwave energy is delivered to tissues via an antenna, with heating concentrated around the antenna’s periphery. Microwave energy can propagate in environments with low thermalelectrical conductivity or high impedance, such as lung tissue, bone, and charred or desiccated tissues. This characteristic allows microwave ablation (MWA) to overcome the limitations of RFA, which has reduced efficacy in such tissue types.^9^ Furthermore, the simultaneous use of multiple MWA antennas enables synergistic thermal effects within the same lesion when placed proximally or simultaneous ablation of separate lesions when positioned distally.^9^

The MWA system comprises three components: the generator, the power delivery system, and antennas. Unlike RFA, the power output from the generator in MWA is unaffected mainly by tissue type. In MWA, there is no significant reduction in power output when operating in high-impedance tissues. The energy generated in the MWA generator is transmitted to the antenna via coaxial cables. However, while flexible and thin-caliber cables are designed to enhance usability, they also introduce disadvantages, such as heating and power loss.^9^ Antennas represent the final component of the system, transferring microwave energy to the tissue. Structurally, antennas consist of an inner conductor, dielectric material, and an outer conductor. The most basic antenna designs include dipole, monopole, and slot antennas. The dipole antenna differs from the monopole design by incorporating a metal block at the distal end of the coaxial cable, leaving a gap for electromagnetic energy emission. The inner and outer conductors are soldered together in slot antennas at the coaxial cable’s end. However, a gap is left near the soldered junction, allowing electromagnetic waves to pass into the tissue. A common challenge in all three antenna designs is generating leaking currents from the antenna back toward the generator.^10^ These currents can cause unintended heating of tissues behind the target area, leading to a comet-tail ablation zone instead of the desired spherical or ellipsoidal shape. Technological advancements have aimed to address this issue.^10^

Compared to RFA, MWA offers distinct advantages due to its underlying physical principles, including achieving larger, more homogeneous ablation zones in a shorter time. However, MWA also has disadvantages, such as less predictable ablation zones, cable heating, the larger calibration of probes, and the creation of elongated and narrow ablation zones.^16^ The indications for MWA are similar to those for other TA techniques and include curative, debulking, or palliative treatments. For colorectal metastases, MWA is recommended for oligometastatic lesions (≤ 4 lesions) smaller than 3 cm.^17^ For HCC, it is indicated for very early-stage (stage 0) and, in selected cases, early-stage (stage A) disease based on the Barcelona Clinic Liver Cancer staging system.^18^

In a review including 11 studies (four randomized and seven observational) and involving 2,169 patients, Spiliotis *et al*. compared the outcomes of MWA and RFA. They reported no significant difference between the two methods in terms of LTP. However, a subgroup analysis of randomized trials in patients with HCC demonstrated lower LTP rates with MWA. No differences were observed between the two techniques regarding complete ablation rates, distant recurrence (DR), or complication rates. The authors emphasized that LTP was the most critical criterion for evaluating the efficacy of ablation techniques.^19^

MWA has been shown to produce more spherical ablation zones, which correspond to larger treatment volumes.^20^ A more spherical ablation zone is expected to ensure complete coverage of the target lesion margin within the ablation area.^21^ Advances in MWA probe design have aimed to achieve this ideal shape. A study by Cazzato *et al*. demonstrated that using multiple MWA antennas (e.g., two antennas for tumors between 2–3 cm, three antennas for tumors > 3 cm) facilitates the creation of ablation zones closer to a spherical shape.^22^

In all ablation methods, including MWA, clinical outcomes are evaluated using parameters such as technical success, the efficacy of the ablation (complete ablation or LTP/local recurrence [LR] in the case of incomplete ablation), complications, and survival (DFS and overall survival [OS]).^19,23^ In a retrospective study by Xu *et al*. involving 142 patients and 294 tumors, technical success was 95.2%, with LTP and DR rates of 15% and 68.3%, respectively. The DFS rates at the first, third, and fifth years were 76%, 33.1%, and 19.5%, respectively, while the OS rates at the same time points were 97.2%, 75.4%, and 50.6%, respectively.^23^ Compared to our study, our three-month DFS rate (95.6%) was significantly better despite our higher LTP rate. Furthermore, tumor sizes were larger in the study by Xu *et al*. (31 ± 13 *vs*. 26.1 ± 13.4 mm).

Pathak *et al*. systematically reviewed 13 studies with 406 patients with colorectal metastases. The OS rates in the first, third, and fifth 5 years were 73%, 30%, and 16%, respectively, while LR rates ranged from 2% to 14%. The minor and major complication rates were 6.7–90.5% and 0–19%, respectively. The authors emphasized that survival rates were higher in patients treated with MWA compared to those receiving palliative chemotherapy alone.^24^ Compared with this review, our patients exhibited a higher recurrence rate (LTP), while the minor and major complication rates were within the range identified.

Leung *et al*. retrospectively analyzed 176 patients and 416 tumors, primarily colorectal metastases (81%), and reported LTP and DR rates of 7.9% and 38%, respectively. The study also demonstrated a significant relationship between tumor size and perivascular location with LR, with the LR rate reaching 33% in tumors larger than 3 cm. In addition, it was emphasized that the LR rate was higher for biliary carcinoma and non-colorectal metastases. The four-year OS rate during the follow-up was reported to be 58% for colorectal metastases and 79% for other pathologies, but no statistically significant differences were observed.^8^ In our study, tumor size was also identified as an independent risk factor for LTP, although a similar relationship between tumor pathology and LTP was not demonstrated. While the cohort in the study by Leung *et al*., mostly consisting of patients with colorectal metastasis, shares similarities with our study, the median tumor size was smaller than ours (10 mm *vs*. 26.1 mm).

To our knowledge, the first clinical study utilizing the MWA antenna with anti-phase technology for ultraspherical ablation was conducted by Blain *et al*.^12^ The authors performed ablation on 87 tumors in 68 patients using the same MWA antenna (Dophi™ M150E, Surgnova, China) as in our study. The lesions had a mean diameter of 17.8 ± 7.9 mm, and the ablation zone measured a maximum axis of 35.6 ± 11 mm. The mean follow-up duration was 10 months, during which local tumor control was observed in 84.7% of the patients who underwent ablation. In contrast, our study demonstrated a DFS rate of 72.8% at 12 months, with a lower tumor control rate than the previous study. Moreover, complications were reported in only two patients in that study (one case of stress ulcer and one of subcapsular hematoma). However, there are several key differences between the two studies, including the median follow-up period (10 months *vs*. 33 months), pre-procedure tumor sizes (median 17.8 cm *vs*. 26.1 cm), MWA procedural parameters (ablation time of 8 ± 4.5 minutes *vs*. 5.2 ± 2.1 minutes), and tumor types (more non-colorectal metastases and an equal number of colorectal metastases and HCC cases in the previous study).

In the prospective study of Zhang *et al*. covering 50 patients and 77 tumors, the majority of tumors were primary lesions [primary: 52, metastatic: 25 (colorectal: 19, non-colorectal: 6) lesions].^11^ Technical success (defined as primary technical efficiency) was shown as 97.4%. Unlike our study, the short and long axes of the ablation zone were measured, and the ablation volume and sphericity index (SI = short axis/long axis) were calculated. When the ablation zone diameters were compared with the manufacturer’s ablation chart and the ex-vivo study of Namakshenas *et al*., it was determined that the long diameters were parallel. In contrast, the short diameters were measured smaller.^14^ It was thought that this situation may be due to the complexity and variable nature of invivo conditions. The parallelism of the ablation zone volumes with the in-vivo study of Blain *et al*. indicated that the antenna provided a predictable ablation zone. The SI value was shown as a mean of 0.77 ± 0.11, and SI was greater than 0.66 in the majority of lesions (86%). This result was interpreted as the ablation zone being “relatively well-rounded, though not perfectly spherical.” In the study of Blain *et al*., where the same formulation was used, the mean SI was 0.78 ± 0.14, and the rate of SI > 0.66 was 82%, showing parallelism.12 No relationship was shown between SI and power and duration. As in our study, attention was paid to lesion locations; factors such as subcapsular location, proximity to the diaphragm and heart, and proximity to major blood vessels or other organs were considered. The clinical follow-up period was short, and multiphasic MRI was performed at 6 weeks, and CT was used in patients with contraindications. The complication rate was stated as 10%, 4% of which were major (liver abscess in 1 patient and liver hemorrhage in 1 patient). The major complication rate is similar to that of our study.

Namakshenas *et al*. investigated the performance of the MWA system used in our study in ex-vivo liver, lung, and kidney ablations.^14^ When the ablation axis measurements were compared with the values in the manufacturer’s chart, the values were generally consistent, especially in low-power settings and single antenna use. Mean SI was 0.95, 0.79, and 0.9 for liver, lung, and kidney, respectively, in single antenna use. It was observed that the SI value approached 1 for 75 W power settings and 10 minutes of ablation in dual antenna use. It was concluded that the ablation axes were predictable except for the highest energy level ablations performed in the lung. It was stated that an almost spherical ablation zone was reached in a single antenna used for the liver and kidney. It was stated that more homogeneous heat distribution was achieved in the tissue in dual antenna use compared to a single antenna. Habert *et al*. performed MWAs on pig liver (50 pieces) and lung (48 pieces) models under CT guidance with the antenna we used in our study and evaluated the ablation dimensions.^13^ In the study, they performed 3, 5, 8, 10 and 15-minute ablations with 50, 75 and 100 W power values. The ablation zone was evaluated 3-dimensionally with contrast-enhanced CT. SI values were determined to be between 0.50-0.80 for the liver and 0.40-0.69 for the lung. Although sphericity was defined as equal to 1, the long and short axes were squared in the SI calculation in this study, and the SI measurements differed from the other studies we mentioned. As a result of the study, it was determined that a shorter ablation time provided better energy efficiency [ablation zone volume (cm^3^)/applied energy (W)], and the ablation zone was more predictable at a 10-minute ablation time.

Our study has certain limitations. Firstly, it was retrospective in design. Secondly, the patient cohort was relatively small and heterogeneous. Due to this heterogeneity, OS data for the patients were not included in the study. Thirdly, there is a notable difference between the HCC group (n = 9) and the metastatic group (n = 83), which could impact statistical analyses when examining subgroups. Lastly, all patients were treated using the same anti-phase technology antenna. While the literature has discussed the potential advantages of this technology over conventional MWA systems, this study did not include any direct comparisons. Further large-scale, comparative, and prospective studies could address these limitations.

The application of anti-phase technology in microwave ablation allows for the formation of more spherical and predictable ablation zones, potentially improving local tumor control and procedural safety in clinical practice.

In conclusion, MWA is a current treatment method for HCC and liver metastases, and technological advancements based on its physical principles are being explored to enhance ablation efficacy. Our study, including the longest followup duration and the largest patient population examining the antenna technology for ultraspherical ablation with anti-phase technology, demonstrated that this treatment had a high technical success rate and acceptable local control and complication rates.
